# The Recent Progresses of Electrodes and Electrolysers for Seawater Electrolysis

**DOI:** 10.3390/nano14030239

**Published:** 2024-01-23

**Authors:** Fan Zhang, Junjie Zhou, Xiaofeng Chen, Shengxiao Zhao, Yayun Zhao, Yulong Tang, Ziqi Tian, Qihao Yang, Evelina Slavcheva, Yichao Lin, Qiuju Zhang

**Affiliations:** 1 Key Laboratory of Far-Shore Wind Power Technology of Zhejiang Province, Hangzhou 311122, China; zhang_f10@hdec.com (F.Z.); chen_xf2@hdec.com (X.C.); zhao_sx@hdec.com (S.Z.); 2 Key Laboratory of Advanced Fuel Cells and Electrolysers Technology of Zhejiang Province, Ningbo Institute of Materials Technology and Engineering, CAS, Ningbo 315201, China; zhaoyayun@nimte.ac.cn (Y.Z.); tangyulong@nimte.ac.cn (Y.T.); tianziqi@nimte.ac.cn (Z.T.); yangqihao@nimte.ac.cn (Q.Y.); 3 Renewable Energy Engineering Institute, Power China Huadong Engineering Corporation Limited, Hangzhou 311122, China; 4 University of Chinese Academy of Sciences, Beijing 100049, China; 5 Qianwan Institute of CNITECH, Ningbo 315201, China; 6 Institute of Electrochemistry and Energy Systems of Bulgaria Academic Science (IEES), Akad. G. Bonchev 10, 1113 Sofia, Bulgaria; eslavcheva@iees.bas.bg

**Keywords:** seawater electrolysis, oxygen evolution reaction, chlorine evolution reaction, electrolyser, electrode

## Abstract

The utilization of renewable energy for hydrogen production presents a promising pathway towards achieving carbon neutrality in energy consumption. Water electrolysis, utilizing pure water, has proven to be a robust technology for clean hydrogen production. Recently, seawater electrolysis has emerged as an attractive alternative due to the limitations of deep-sea regions imposed by the transmission capacity of long-distance undersea cables. However, seawater electrolysis faces several challenges, including the slow kinetics of the oxygen evolution reaction (OER), the competing chlorine evolution reaction (CER) processes, electrode degradation caused by chloride ions, and the formation of precipitates on the cathode. The electrode and catalyst materials are corroded by the Cl^−^ under long-term operations. Numerous efforts have been made to address these issues arising from impurities in the seawater. This review focuses on recent progress in developing high-performance electrodes and electrolyser designs for efficient seawater electrolysis. Its aim is to provide a systematic and insightful introduction and discussion on seawater electrolysers and electrodes with the hope of promoting the utilization of offshore renewable energy sources through seawater electrolysis.

## 1. Introduction

The environmental pollution associated with the excessive consumption of fossil fuels has driven efforts to utilize renewable clean energy. Hydrogen is regarded as the cleanest renewable energy to achieve carbon-neutral goals, due to its high energy density of 142.35 kJ kg^−1^, abundant storage in nature, and no pollution with the unique product of water [1]. Currently, the industrial methods of producing hydrogen mainly include methane steam reforming [2,3], alcohol cracking [4,5], coal gasification [6,7], and water electrolysis [8,9]. Due to the energy loss during the transmission and usage process, the CO_2_ emission is many times higher than the direct utilization of electricity with fossil fuel as the energy source as shown in Table 1. Compared to the hydrogen produced by burning fossil fuels, the hydrogen obtained from water electrolysis is called clean hydrogen because no carbon footprint is involved.

Compared to pure water electrolysis, seawater electrolysis offers distinct advantages in terms of cost and operational efficiency. Utilizing pure water in electrolysis typically requires the incorporation of supplementary water purification systems, leading to increased energy consumption and financial expenditures. The conventional seawater desalination methods are also energy-intensive. Without the purification systems, the electrolyser could be designed more compactly, resulting in lower capital costs and integrated engineering challenges. Additionally, freshwater is usually a valuable resource and many people face the problem of water security. Renewable energy power is generated from wind or solar energy, of which the utilization is seriously limited by its intermittent problem. Therefore, electrolysis is considered an important method of converting renewable energy to fine chemicals such as hydrogen [10], ammonia [11] and other hydrocarbons [12]. Industrial hydrogen production by pure water electrolysis in alkaline conditions has been realized. With abundant renewable electricity generated in deep-sea regions, on-site hydrogen production through seawater electrolysis gradually becomes more and more attractive due to limitations by the transmission capacity of long-distance undersea cables. For example, the maximum transmission distance for a 220 kV AC undersea cable is approximately 80 km at the 300 MW power level [13].

In past decades, significant progress has been achieved in improving the performance and stability of pure water electrolysis [14]. However, seawater contains more complicated ions than pure water, including 3.5 wt% Na^+^ and Cl^−^ along with minor amounts of Ca^2+^, Mg^2+^, and Br^−^. The main challenge of seawater electrolysis is how to reduce the impact of these impurity ions on electrodes and catalysts to prevent surface Cl^−^ corrosion and elongate their lifetime. The catalyst of NiFe alloys encapsulated into a defective graphene layer is designed, which requires only 276 mV overpotential in alkalized seawater to reach 100 mA cm^−2^ and lasts for 2000 h continuously [15]. Lu et al. developed a strategy in which AgCl on the anode surface could repel free Cl^−^ through a strong common-ion repulsive effect and lead to the electrode’s stability over 5000 h [16]. These elongated stable catalysts make it possible to realize seawater electrolysis commercially.

With the rapid research progress on developing stable electrodes or efficient catalysts for seawater electrolysis, large-scale hydrogen production becomes potentially applicable. Currently, the alkaline water electrolysis (AWE) electrolyser develops rapidly, but it contains many drawbacks, such as low energy efficiency, slow response speed and large size. These disadvantages make it difficult for AWE to satisfy the requirements for offshore seawater electrolysis [17,18]. Therefore, it is necessary to develop other types of electrolysers for seawater electrolysis. Several types of electrolysers have been designed according to different membrane types, electrolytes and electrolysis conditions, involving proton exchange membrane water electrolysis [19,20] (PEMWE), anion exchange membrane water electrolysis [21,22,23] (AEMWE), solid oxide electrolysis cell [24,25] (SOEC) and other electrolysers [26,27,28].

To provide a systematic and insightful introduction and discussion on seawater electrolysers, we review recent advancements in designing electrodes and electrolysers for seawater electrolysis. Recently, electrocatalysts and electrolysers have been introduced in some literatures [29,30,31]. This review focuses on the long-term stable operation of seawater splitting, considering the practical situations. We begin by introducing the reaction mechanism of seawater electrolysis on electrodes and the recent development of cathodes and anodes. Subsequently, we discuss the progress in designing seawater electrolysers, with a focus on four types of electrolysers. Finally, we propose research challenges and issues related to seawater electrolysis. This review aims to promote the development of offshore renewable energy sources through seawater electrolysis.

## 2. Water Splitting Reactions and Design Principles of Electrodes

The overall reaction of seawater electrolysis is the same as that of pure water splitting, as shown in Equation (1).

Overall reaction: 2H_2_O → 2H_2_ + O_2_ (1)

The reaction consists of two half-reactions: the hydrogen evolution reaction (HER) on the cathode and the oxygen evolution reaction (OER) on the anode. The expressions under different pH conditions are presented in Equations (2)–(5). In acidic media, the two half-reactions are illustrated by the following two equations.

HER: 2H^+^ + 2e^−^ → H_2_ (2)

OER: 2H_2_O → 4H^+^ + O_2_ + 4e^−^ (3)

In neutral or alkaline media, the two half-reactions are expressed by Equations (4) and (5).

HER: 2H_2_O + 4e^−^ → H_2_ + 2OH^−^ (4)

OER: 4OH^−^ → O_2_ + 2H_2_O + 4e^−^ (5)

CER: 2Cl^−^ → Cl_2_ + 2e^−^ (6)

In seawater electrolysis, the chlorine evolution reaction (CER) is also a competitive process with OER, producing Cl_2_ in Equation (6). The detailed mechanisms and design principles of electrodes are discussed in the next section.

### 2.1. HER and OER Mechanism

HER is a classical 2e^−^ process, with the reaction efficiency dependent upon H^+^ concentration at the reaction interface. Under different pH conditions, HER typically involves three steps:

Volmer step: H_3_O^+^ + e^−^ + M → MH_ads_ + H_2_O (7)

Heyrovsky step: MH_ads_ + H^+^ + e^−^ → H_2_ + M (8)

Tafel step: MH_ads_ + MH_ads_ → H_2_ + 2M (9)

**Figure 1:**
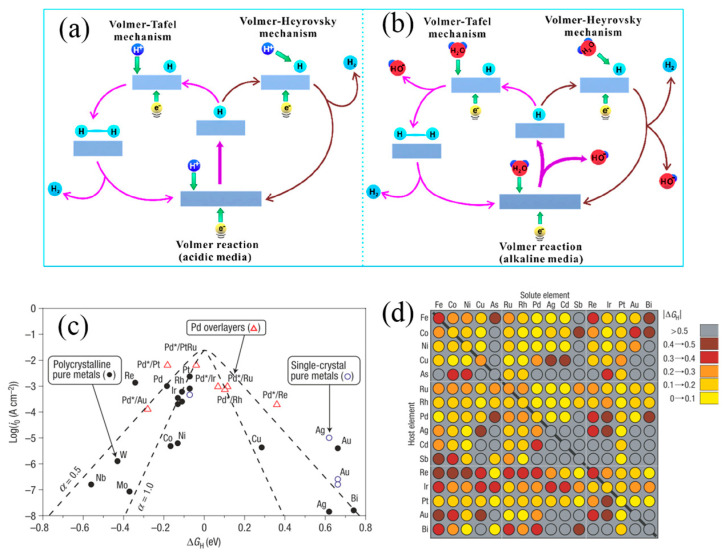
Mechanism of HER in acidic (**a**) and alkaline (**b**) solutions [32]. Copyright 2020, American Chemical Society. (**c**) Volcano plot for the HER for various pure metals and metal overlayers. (**d**) Computational high−throughput screening for |G_H_| on 256 pure metals and surface alloys [33]. Copyright 2006, Nature.

As shown in Figure 1a,b, H_3_O^+^ serves as the proton source in the Volmer step, combining with electrons at the catalysts’ active sites and resulting in the formation of adsorbed active hydrogen atoms (MH_ads_) [32]. The difference in the subsequent H_2_ generation step is that an activated MH_ads_ accepts H^+^ and e^−^ to form H_2_ in the Heyrovsky step, while two activated MH_ads_ combine together to produce H_2_ in the Tafel step. The Tafel slope is a vital indicator of evaluating the catalyst performance because it allows us to infer the mechanism occurring on the catalyst surface based on its value. Considering these three steps, in the HER, the formation of adsorbed active H^+^ on the electrode surface is a fundamental characteristic. Norskov et al. proposed that the interaction between H atoms and metals affects the activation energy of the H^+^ discharge process, as shown in Figure 1c. The ‘volcano effect’ plot indicates that Pt-group elements with moderate M-H_ads_ bond strength own the best HER catalytic performance [33]. However, it is observed that Pt is not located at the volcano’s peak, indicating HER catalytic activity could be further enhanced.

Compared to the 2e^−^ process of HER, four electrons (4e^−^) transferring OER is much more sluggish. The reported OER mechanisms mainly include the adsorption evolution mechanism (AEM), the lattice oxygen-mediated mechanism (LOM) and the oxidation path mechanism (OPM).

**Figure 2:**
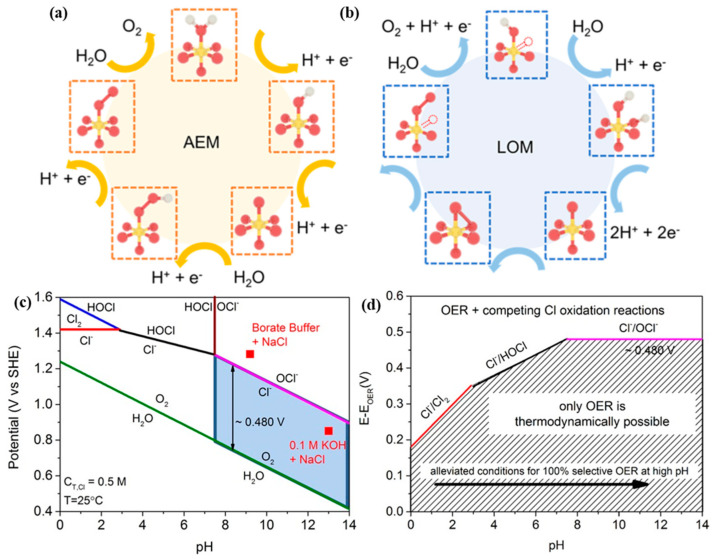
Mechanism of OER consists of (**a**) adsorbate evolution mechanism (AEM), (**b**) lattice oxygen mechanism (LOM) [34]. Copyright 2023, American Chemical Society. (**c**) Pourbaix diagram for artificial seawater model. (**d**) Maximum allowed overpotential of OER to ensure 100% selective in seawater splitting [35]. Copyright 2016, Wiley.

AEM for the anodic OER is represented as shown in Equations (10)–(13) and Figure 2a, where the first two steps involve the adsorption intermediates of OH_ads_ and O_ads_ at the active sites of the catalyst [34]. The adsorbed O_ads_ intermediate further transforms into OOH_ads_, which subsequently reacts with OH^−^ to produce O_2_.

M + OH^−^ → MOH_ads_ + e^−^ (10)

MOH_ads_ + OH^−^ → MO_ads_ + H_2_O + e^−^ (11)

MO_ads_ + OH^−^ → MOOH_ads_ + e^−^ (12)

MOOH_ads_ + OH^−^ → O_2_ + M + H_2_O + e^−^ (13)

Similar to AEM, the metal site of LOM is the active centre in OER process, which is shown in Equations (14)–(17) and Figure 2b [34]. The initial step is the formation of an adsorbed *OH on the metal site. Then the adsorbed *OH reacts with the lattice-O to produce *OOH, which departs from the catalyst surface in the form of O_2_ molecules after reacting with OH^−^. Cho et al. theoretically calculated the overpotentials of perovskite materials under AEM and LOM [36]. They found that the theoretical overpotential of the rate-limiting step is only 0.17–0.41 V in LOM, significantly lower than the predictions of AEM, indicating that LOM is more efficient.

OH* → V_o_ + *OO + H^+^ + e^−^ (14)

H_2_O(l) + V_o_ + *OO → O_2_(g) + V_o_ + *OH + H^+^ + e^−^ (15)

(Vo + OH*) + H_2_O(l) → (HO-site* + *OO) + H^+^ + e^−^ (16)

(*OO + HO-site*) → OH* + H^+^ + e^−^ (17)

OPM is illustrated in Equations (18)–(20), which could be summarized in three steps, involving the *OH adsorption, reaction with OH^−^ to produce *O-intermediate and O_2_-production by coupling two adsorbed *O and then desorption from the catalyst surface to release the active site. Unlike LOM, OPM does not require oxygen vacancies to participate in the reaction, and its reaction activity is only related to *OH and *O, demonstrating superior activity and stability. However, it is difficult to directly couple these two *O species to form O_2_ in the last step in OPM, due to stringent spatial constraints on the metal active sites.

2OH^−^ + 2M → 2MOH_ads_ +2e^−^ (18)

2MOH_ads_ + 2OH^−^ → 2MO_ads_ + 2H_2_O + 2e^−^ (19)

2MO_ads_ → O_2_ + 2M (20)

### 2.2. Design Principles of Cathode and Anode

The development of cathodic HER catalysts in seawater is correlated with three aspects. Firstly, it is necessary to reduce the cost of seawater electrolysis by decreasing the load of Pt-group precious metals or developing efficient nonprecious metal catalysts. Secondly, the precipitates of Ca^2+^ and Mg^2+^ in seawater should be diminished, as they result in the catalysts’ deactivation and high energy consumption. Finally, anticorrosion catalysts should be designed to elongate the lifetime, since corrosion of Cl^−^ and Cl^−^ oxidation products, such as Cl_2_, HClO, and ClO^−^ has a negative effect on the stability of catalyst. To address the first issue, three main strategies are developed, including reducing the surface size of the catalyst to enhance its catalytic performance, modifying the Pt-based catalysts to enhance the catalytic activity, and using metal alloys or transition metal catalysts to reduce the content of Pt-based metals. For the second issue, the common strategy is to modify the microenvironment of the catalyst surface to resist the Ca^2+^ and Mg^2+^, such as incorporating some cationic groups or using Lewis acidic substances as carriers. For the last issue, it is important to design the anode electrocatalyst with high OER selectivity but avoiding the Cl^−^ oxidation products.

**Figure 3:**
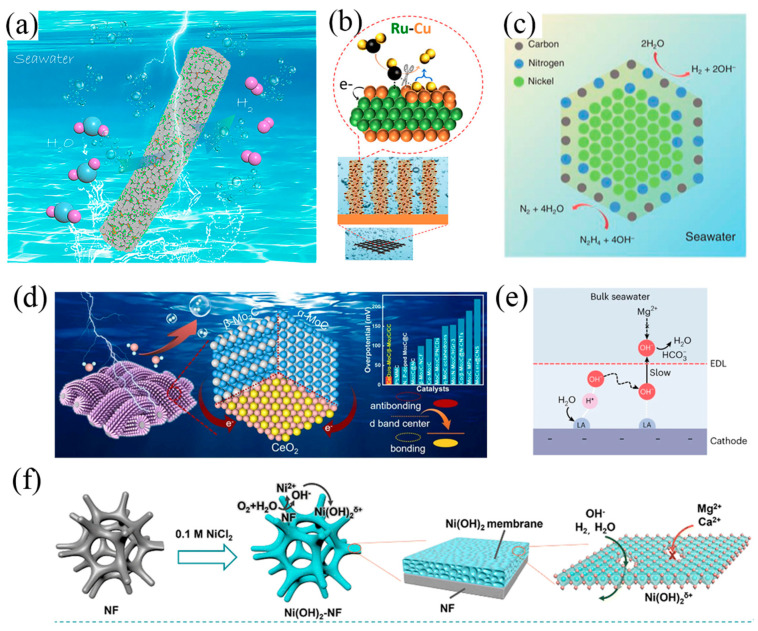
(**a**) Diagram of Pt−Ni@NiMoN electrocatalyst for seawater HER [37]. Copyright 2023, Royal Society of Chemistry. (**b**) Diagram of Ru−Cu nano-heterostructures for efficient HER [15]. Copyright 2023, American Chemical Society. (**c**) Proposed structure of the Ni−SN@C catalyst [49]. Copyright 2021, Wiley. (**d**) Scheme of hetero-structured CeO_2_/α−MoC/β−Mo_2_C electrocatalyst [44]. Copyright 2022, Elsevier. (**e**) Scheme of Lewis acid to facilitate HER and prevent precipitate formation [62]. Copyright 2023, Nature. (**f**) Scheme of Ni(OH)_2_ membrane grown in situ to repel Cl^−^ [63]. Copyright 2023, Royal Society of Chemistry.

Until now, various methods have been developed for improving HER performance, including alloy [37,38,39], doping [40,41], selenization [42,43], carbonization [44,45,46,47,48], nitridation [49,50,51,52], etc. The design of cathode electrocatalysts has been introduced in many other reviews [53,54,55]; herein, we only provide some representative work. One of the effective strategies is reducing the use of precious metals while maintaining a high HER current. Yang et al. prepared a Pt-Ni alloy with hierarchically heterogeneous Pt–Ni@NiMoN/NF structure (Figure 3a) [37]. An ultra-low Pt loading (0.07 wt%) exhibited outstanding HER catalytic activity in 1 M KOH and real seawater, showing small overpotentials and long-term stability at high current densities (≥400 mA cm^−2^). The X-ray photoelectron spectroscopy (XPS) results suggested that Pt acts as an electron donor with charge transfer to Ni and Mo. The Ni K-edge X-ray absorption near-edge structure (XANES) spectra and extended X-ray absorption fine structure (EXAFS) both indicated electronic interactions between Ni and surrounding atoms. Doping is another way to improve HER performance with low noble metal content. Fu’s group compared the possible HER catalytic mechanism by *operando* EIS technique and found that the phase angle of bimetal doped NiSe_2_ catalyst named Ru,W-NiSe_2_ displayed a relatively large downward trend in the low frequency than single metal doped NiSe_2_ as the bias potential increased [56]. This phenomenon attributed to Ru,W-NiSe_2_ have a faster charge transfer process at the catalyst-electrolyte interface, which is the rate-determining rate rather than the electron transfer step. A similar view has been presented in another study, where the charge transfer resistance of 3D Ni-Mo (0.6 Ω) is 16 times lower than other 2D Ni-Mo (10.3 Ω), which showed the fast transport of ions and facilitates the H* desorption process [57]. Manna et al. designed a hierarchical electrode composed of amorphous-TiO_2_/Cu nanorods decorated with Ru-Cu nano-heterostructures (Figure 3b) [15]. Low loading of Ru (52 μg cm^−2^) exhibited a small HER overpotential of 74 mV at 200 mA cm^−2^. The XANES record for Ru K-edge clearly shows that Ru species within the electrode are not in RuO_2_ form. The corresponding Fourier Transform of the extended X-ray absorption fine structure (FT-EXAFS) exhibits a prominent main peak at ca. 2.4 Å, which is consistent with Ru−Ru bonds of metallic Ru. These *operando* characterizations were applied to the study of the changes in catalyst surfaces/interfaces [58,59,60]. Except for Pt-group elements as shown in Figure 1d, nonprecious TMs are also ideal substituted catalysts with lower cost. Ren et al. designed a sandwich-like NiCoN|Ni_x_P|NiCoN microsheet array catalyst [61]. The chlorine-corrosion resistance was realized by the inner Ni_x_P microsheet arrays. The NiCoN coating structure provided a large surface area with abundant active sites, improved intrinsic activity of every active site, and high electrical conductivity for efficient charge transfer. Zhang fabricated an outperforming Mo carbide-based electrocatalysts called CeO_2_/α-MoC/β-Mo_2_C (Figure 3d) [44]. It displayed a low overpotentials of 29 mV at 10 mA cm^−2^ in alkaline seawater, which showed the best performance among the reported Mo carbide-based electrocatalyst. The Ni-SN@C catalyst exhibited excellent HER activity in alkaline seawater, which was synthesized by an unsaturated nitriding process to encapsulate the unsaturated nickel surface nitride (Figure 2c). A low overpotential of 23 mV was achieved at a current density of 10 mA cm^−2^ [49].

The cathode faces an additional issue, where significant precipitation of Mg^2+^ and Ca^2+^ occurs on the HER electrode, leading to blocking of the diaphragm and deactivation of the catalysts [64]. To prevent the cathode from precipitation, a robust Lewis acid layer was incorporated onto the catalyst’s surface (Figure 3e), which served to split water molecules and capture in situ generated OH^−^ anions [62]. Due to the robust affinity between OH^−^ and the Lewis acid layer, it significantly reduces the OH^−^ concentration within the electrical double layer. Any desorbed OH^−^ ions are neutralized by buffer ions in seawater, maintaining a pH of around 8.5, which is lower than the precipitation threshold of pH = 9.5. The flow-type seawater electrolyser demonstrated excellent stability, withstanding 100 h at 500 mA cm^−2^ and achieving an industrially required current density of 1.0 A cm^−2^ at 1.87 V and 60 °C [62]. In addition to the protection by the Lewis acid layer, Wang et al. reported a charged Ni(OH)_2_ nanofiltration membrane (Figure 3f) in situ grown on Ni foam as an anti-precipitation layer. Simulations and experiments revealed that the positively charged Ni(OH)_2_ membrane with nanometer-scale cracks evidently hindered the transfer of Mg^2+^ and Ca^2+^ while rapidly transferring OH^−^ and H_2_O. The Ni(OH)_2_ membrane decorated seawater HER electrode reduced precipitation by about 98.3% and exhibited high activity and stability [63]. Numerous studies have investigated the alkaline OER mechanism using noble metal catalysts [65]. Among these metals, Ruthenium (Ru) based oxides show a relatively low overpotential and high stability in the OER [66]. One strategy for catalyst design is to reduce the loading of Ru or replace it with nonprecious metals. For example, the use of a nickel-iron (NiFe) layer double hydroxide (LDH) has been found to exhibit comparable OER performance. Many efforts have been devoted to further improve OER activity of NiFe-LDH, such as increasing the high selectivity of OER by doping, heterostructure, defect engineering, crystal structure and electron transport path reported in many recent reviews [67,68,69,70].

The Cl^−^ corrosion at the anode is a main challenge of the long-term operation. The presence of Cl^−^ ions (~0.5 M) in seawater induces CER competition with the OER at the anode. The Pourbaix diagram for the artificial seawater model is shown in the Figure 2c [35]. In acidic conditions at pH = 0, the theoretical overpotential for OER (1.23 V vs. SHE) is 130 mV lower than that for CER (1.36 V vs. SHE). Since OER involves a 4e^−^ transfer process, CER is kinetically favourable due to its 2e^−^ transfer process. Despite Cl_2_ being a high-value chemical, it suffers toxic character and challenges of storage and transportation. In an alkaline environment, another possible reaction is the formation of hypochlorite ions (ClO^−^) by oxidizing Cl^−^ ions. The generation of ClO^−^ also competes with OER reaction, with its initial potential 480 mV higher than that of OER (Figure 2d). To prevent the formation of ClO^−^, the overpotential of the OER catalyst under actual industrial application current density (approximately 1 A cm^−2^) requires being less than 480 mV. This would effectively suppress the unwanted ClO^−^ generation. Obviously, OER possesses faster reaction kinetics under alkaline conditions due to more favourable OH-intermediates.

**Figure 4:**
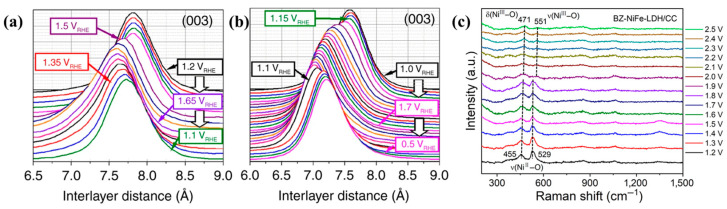
Waterfallplot of normalized and background-subtracted (003) peak obtained during in situ WAXS in 0.1 M KOH and potential steps for (**a**) NiFe LDH and (**b**) CoFe LDH [71]. Copyright 2022, Tsinghua University Press. (**c**) in situ Raman spectra collected for BZ-NiFe-LDH/CC at different potential [72]. Copyright 2020, Nature.

The physicochemical properties of the catalyst’s surface, the reactant and the electrolyte determine the performance. To understand the catalytic mechanization, characterizations are needed to analyze the electrocatalyst, such as X-ray photoelectron spectroscopy (XPS), X-ray absorption spectroscopy (XAS), scanning probe microscopy (SPM), wide-angle X-ray diffraction (WAXRD), auger electron spectroscopy (AES), transmission electron microscope (TEM). Generally, these techniques can only be applied under ex situ conditions and cannot fully capture the changes of catalysts in real chemical reactions. To observe the seawater splitting processes clearly, in situ/*operando* characterizations were utilized. To investigate the catalyst’s structural changes, in situ wide-angle X-ray scattering (WAXS) is employed. Dionigi et al. tracked structural transformations with *operando* WAXS and found under applied anodic potentials, the (003) diffraction peak of MFe (M = Ni and Fe) LDH shifted to a shorter interlayer space as shown in the Figure 4a,b [71]. It meant the MFe LDH transform from the as-prepared α-phase to the active γ-phase. To observe the phase evolution of the catalysts, the *operando* Raman technique is utilized. Zhang et al. reported that BZ-NiFe-LDH/CC (benzoate anions intercalated NiFe LDH nanosheet array on carbon cloth) exhibited a redshift trend for the peaks at 455 cm^−1^ and 529 cm^−1^ by combining the electrocatalytic measurements with the *operando* Raman characterizations as shown in the Figure 4c [72]. The decreasing peak densities evidenced the existence of δ(Ni^III^-O) and (γ-NiOOH), which are the active sites of the OER in seawater. In addition to the above-mentioned techniques, more and more characterizations are proposed, such as the laser-induced current transient technique [73,74,75], synchrotron-based X-ray spectroscopies [65], and so on.

**Figure 5:**
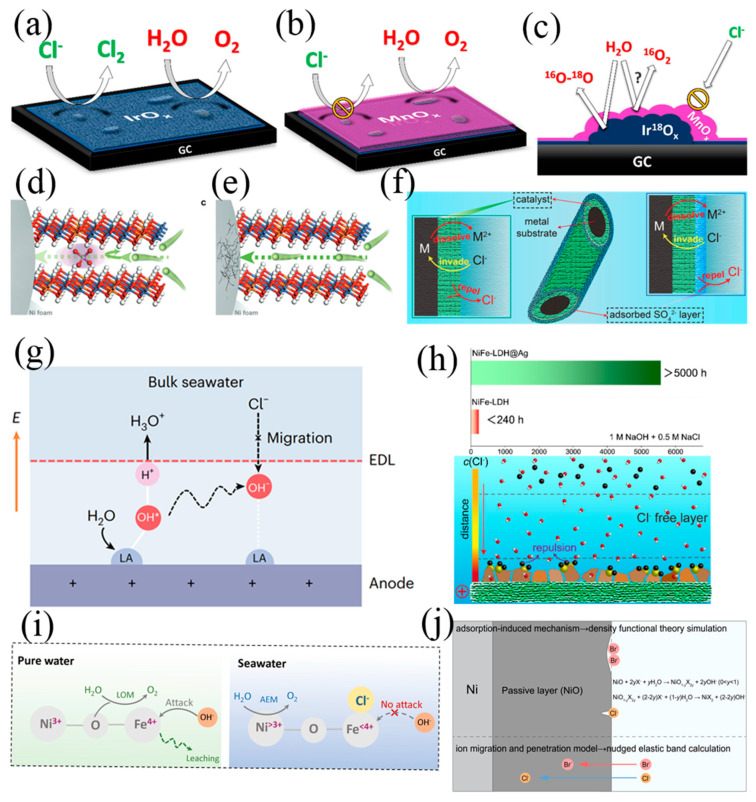
(**a**–**c**) The MnO_x_ on top of the IrO_x_ layer, blocking ClOR by preventing Cl^−^ from reaching the IrO_x_ underneath [77]. Copyright 2018, American Chemical Society. Scheme of Cl^−^ crossing through the NiFe−LDH with the PO_4_^3−^ anion (**d**) and without any anions intercalation (**e**) [79], Copyright 2023, Wiley. (**f**) Catalysts optimization (**left**) and electrolyte optimization (**right**) to protect the metal substrate from Cl^−^ corrosion by adsorbing SO_4_^2−^ layer [81]. Copyright 2021, Wiley. (**g**) Lewis acid on the anode facilitates OER and inhibits chlorine chemistry [62]. Copyright 2023, Nature. (**h**) The Schematic illustration of in situ AgCl effectively repel free Cl^−^ through strong common-ion repulsive effect [16]. Copyright 2023, Wiley. (**i**) The schematic illustration for structure evolution and OER mechanism transfer in alkaline pure water and alkaline seawater [82]. Copyright 2023, Wiley. (**j**) The schematic diagram of the two pitting initiation mechanisms [83]. Copyright 2023, Nature.

Some progress has been achieved in preventing Cl^−^ corrosion in seawater electrolysis. Constructing Cl^−^ blocking layers on the catalyst is found to be viable to circumvent the competition reaction and corrosion in Cl^−^ abundant seawater electrolysis. In 1980, Bennett firstly found the electrocatalyst coated with MnO_2_ at the anode can achieve high O_2_ selectivity. After that, many researchers verified this phenomenon [76]. Koper et al. deposited a MnO_x_ layer on the IrO_x_/glassy carbon and found that the MnO_x_ layer decreased the selectivity of ClOR from 86% to 7%. As shown in Figure 5a–c, the MnO_x_ layer served as Cl^−^ repelling layers rather than the OER active sites [77]. Maccato et al. developed a Co_3_O_4_ catalyst decorated with MnO_2_ to work in alkaline seawater for 6 months [78].

Integrating the catalyst with anti-Cl^−^ layers can increase the overpotential of ClOR on the electrode surface, which is favourable to achieving a high Faradaic efficiency for OER. Sun et al. reported an anti-corrosion strategy of PO_4_^3−^ intercalation in NiFe-LDH (Figure 5d,e), in which the highly negative-charged PO_4_^3−^ in the interlayers could prevent Ni substrate from Cl^−^ corrosion by electrostatic repulsion [79]. PO_4_^3−^ effectively hindered the migration of Cl^−^ between the interlayers of NiFe-LDH, resulting in a significantly longer lifetime compared to pristine NiFe-LDH. The improvement of stability was attributed to the inhibition effect of Cl^−^ passing through the interlayers of NiFe-LDH, leading to the protection of Ni substrate. Pan et al. developed a sulfate ion (SO_4_^2−^) modulated strategy to boost OER activity of Nickel-iron oxy-hydroxides (NiFeOOH) [80]. The experimental and theoretical investigations demonstrated the dual effect of SO_4_^2−^ on improving OER performances. SO_4_^2−^ leaching was favourable for the electrochemical reconstruction to form active NiFeOOH under OER conditions. Simultaneously, the residual SO_4_^2−^ surface could stabilize the intermediate of OOH* and enhance OER performance. As expected, the optimized electrocatalyst delivered an ultralow overpotential of 234 mV to reach the current density of 50 mA cm^−2^ and a high stability for more than 100 h. Lu et al. proposed that the existence of SO_4_^2−^ in electrolytes could effectively alleviate the Cl^−^ corrosion on anode in alkaline seawater electrolysis (Figure 5f), resulting in significantly enhanced operation stability of the anode [81]. This enhancement was attributed to the preferential adsorption of additive SO_4_^2−^ on anode surface, which repelled Cl^−^ in bulk phase by electrostatic repulsion. Due to the repulsive effect of additive SO_4_^2−^, the active NiFe-LDH nanoarrays/Ni foam anode is stable for 500–1000 h corresponding to the simulated and natural seawater. It is believed that the repulsive effect of electrolyte additives would be generally applicable for other active OER electrodes and favour the scale-up of alkaline seawater electrolysis.

The protective layer attained by incorporating a robust Lewis acid layer onto the catalysts’ surface could also reduce Cl^−^ corrosion on the anode, because the Lewis acid layer splits H_2_O and captures in situ generated OH^−^ while repels in situ Cl^−^ (Figure 5g) [62]. Lu et al. proposed a novel surface Cl^−^ fixation method (Figure 5h) by loading silver nanoparticles onto the catalyst surface to in situ form AgCl nanoparticles [16]. This method achieved specific repulsion of Cl^−^ within the double layer of the electrode surface, and, hence, significantly enhanced the stability of the anode in seawater electrolysis. The optimized NiFe-LDH@Ag electrode, working at a current density of 400 mA cm^−2^ in a 1 M NaOH + 0.5 M NaCl or 1 M NaOH + seawater electrolyte, could last for over 5000 and 2500 h, respectively. The chlorine was found to not always harm the seawater splitting. The Popov group simulated and reported that chlorine around the surface with H_2_O adsorbate could trigger the dehydrogenation of a water molecule from the thermal effect and photoexcitation [84]. Qiao et al. offered a different explanation of the impact of Cl^−^ on electrocatalysts, stating that the adsorption of Cl^−^ on the desired Fe site was believed to suppress Fe leaching (Figure 5i), and, hence, created more OER-active Ni sites to improve the catalyst’s long-term stability and activity simultaneously [82].

Bromide ions (Br^−^) were found to pose a greater threat to Ni-based anodes with a faster corrosion rate [83]. The corrosion resistance of Ni substrates in solutions containing Br^−^ was inferior to that of those containing chloride ions (Cl^−^), which was elucidated by the cyclic polarization curves (Figure 5j). The in situ electrochemical characterization revealed that Cl^−^ caused localized corrosion of the substrate, forming narrow and deep pits. In contrast, Br^−^ caused broad-area corrosion, creating shallow and wide pits. An in-depth analysis of the mechanism showed that Cl^−^ had a lower diffusion barrier, allowing it to easily penetrate the passivation layer of the substrate and cause corrosion, while Br^−^ reacted with the passivation layer with lower free energy, leading to rapid corrosion at multiple sites. Additionally, for Ni-based electrodes with a catalyst surface (such as NiFe-LDH), Br^−^ caused large-scale delamination of the catalyst layer, leading to a rapid decline in OER performance.

## 3. Seawater Splitting Electrolysers

To date, the predominant method for seawater electrolysis is AWE, which is presently in the pilot stage. Other technologies, such as AEMWE, PEMWE, and SOEC, have achieved significant progress in the laboratory [85]. The configurations and work conditions of these electrolysers were compared in Table 2. In this section, we not only introduce the design and work principles of these electrolysers and their advanced characteristics, but also offer insights into current progress and prospective views about these technologies.

### 3.1. AWE Electrolyser

**Figure 6:**
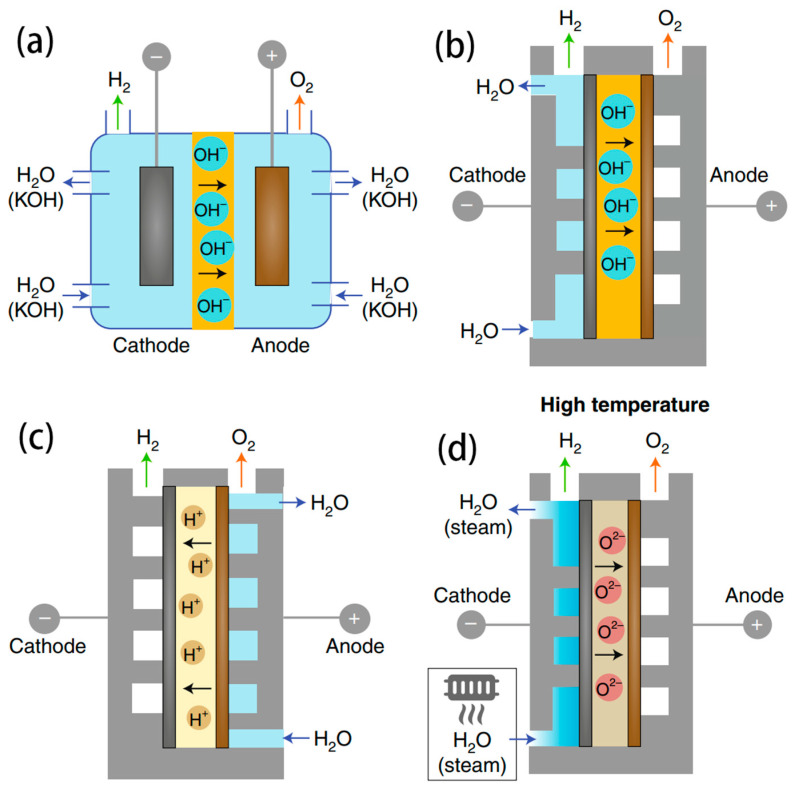
Configuration for seawater electrolysis: (**a**) alkaline water electrolysis (AWE) electrolyser; (**b**) anion exchange membrane water electrolysis (AEMWE) electrolyser; (**c**) proton exchange membrane water electrolysis (PEMWE) electrolyser; (**d**) solid oxide electrolysis cell (SEOC) [85]. Copyright 2023, Nature.

AWE is a mature technology that has already been used to produce large-scale hydrogen production from pure water [86]. The schematic representation of the electrolysis cell is depicted in Figure 6a. where AWE typically employs a porous membrane to segregate the cathode from the anode, with each compartment undergoing alkalization through a 30–40 wt% KOH solution. A commonly used membrane in AWE is the Zirfon membrane, which is more physically robust and less susceptible to blockages compared to PEM/AEM. During the electrolysis process, OH^−^ migrates to the anode through the diaphragm to be oxidized to O_2_. AWE possesses numerous advantages, including a simple structure, scalability, large single-cell hydrogen production capacity, and low capital investment. This makes it the most applicable for seawater electrolysis to realize industrial-scale hydrogen production. Until now, the AWE electrolyser cost has been significantly reduced, with the development of electrodes with low-precious metals and improved performance in preventing corrosion. The widely used electrodes are Ni-based, Co-based and stainless-steel electrodes.

**Figure 7:**
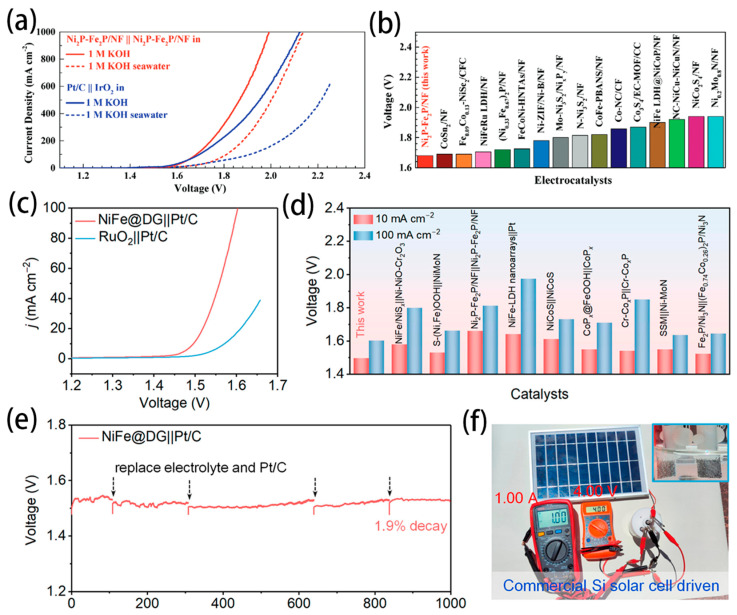
(**a**) Overall water/seawater splitting performance of Ni_2_P-Fe_2_P/NF and the Pt/C||IrO_2_ pair in 1 M KOH and 1 M KOH seawater. (**b**) Comparison of the voltages at a current density of 100 mA cm^−2^ for seawater splitting between Ni_2_P-Fe_2_P/NF and other electrocatalysts [87]. Copyright 2020, Wiley. (**c**) LSV curves of the NiFe@DG||Pt/C and RuO_2_||Pt/C electrolysers. (**d**) Comparison of voltages at 10 and 100 mA cm^−2^ for the NiFe@DG||Pt/C pair with recently reported catalysts. (**e**) Durability measurement of the AWE electrolyser at 10 mA cm^−2^. (**f**) Photograph of seawater electrolysis driven by a commercial Si solar cell [88]. Copyright 2023, American Chemical Society.

A main challenge in seawater electrolysis is the corrosion on electrodes caused by Cl^−^ ions. Researchers have discovered that modifying the electrode surface with a negatively charged group can effectively reduce corrosion and degradation. This modification significantly extends the lifetime of the carriers and enhances the stability of the electrodes. A heterogeneous Ni_2_P-Fe_2_P micro-sheet has been synthesized by directly soaking Ni foam in HCl and Fe(NO_3_)_3_ solution followed by phosphidation [87]. The hydrophilic surface was more applicable for OER rather than CER by enhancing the corrosion resistance. This self-supported Ni_2_P-Fe_2_P electrocatalyst exhibited excellent catalytic activity toward overall water electrolysis, only requiring low voltages of 1.682 and 1.865 V to reach large current densities of 100 and 500 mA cm^−2^ in 1M KOH, respectively (Figure 7a,b). Fei and coworkers reported that the NiFe alloy encapsulated within defective graphene layers (NiFe@DG) was prepared by a flashing heating method [88]. The presence of defective graphene coating on the electrode surface created a built-in electric field, which protected against chloride ions at the electrode/electrolyte interface. The AWE electrolyser assembled with NiFe@DG and Pt/C electrodes needs voltages of only 1.496 and 1.602 V to afford current densities of 10 and 100 mA cm^−2^, respectively. It is much lower than the benchmark RuO_2_||Pt/C pair and other reported electrodes (Figure 7c,d). Figure 7e shows that the NiFe@DG||Pt/C electrolyser displays high durability with a 28 mV voltage fluctuation (1.9% decay) over the 1000 h continuous electrolysis. This electrolyser could be driven by the commercial Si solar cell, as shown in Figure 7f, indicating its feasibility and potential.

To date, direct seawater electrolysis methods have faced certain limitations, such as ClOR competition, halide corrosion, and hydroxide precipitation. Consequently, by drawing upon the paradigm of classical pure water electrolysis, the process of filtering seawater into pure water prior to electrolysis obviates these constraints. This approach also serves to enhance the service lifetime of both the catalyst and the electrode effectively.

**Figure 8:**
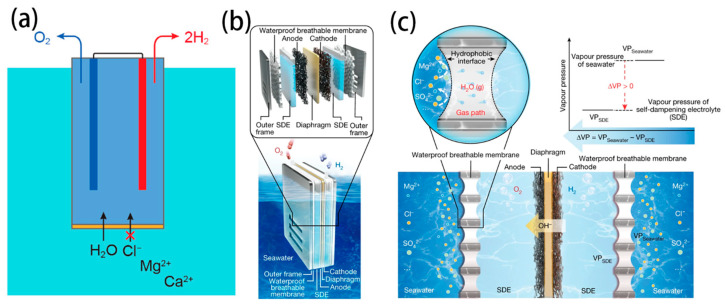
(**a**) Schematic of a forward-osmosis water-splitting cell [28]. Copyright 2022, American Chemical Society. (**b**) Schematic diagram of a typical seawater electrolysis system. (**c**) The liquid–gas–liquid phase transition-based migration mechanism of the water purification and migration process and the driving force [27]. Copyright 2022, American Chemical Society.

Recently, Nocera et al. reported a unique direct seawater electrolysis technology, as shown in Figure 8a, which couples forward osmosis (FO) membrane with water splitting to achieve continuous hydrogen production from impure water sources [27]. In this configuration, a 0.8 M NaPi solution and a 0.6 M NaCl solution served as the electrolyte and artificial seawater, respectively. The cellulose acetate membrane (CAM) was chosen to prevent the AWE electrolyser from contacting the seawater directly. Due to the concentration gradient between the two sides of the membrane, H_2_O can forward osmosis into the AWE electrolyser spontaneously. Water splitting to produce H_2_ and O_2_ drove an outflux of water and generated a concentration gradient, which was then balanced by an influx of water provided by the FO membrane. It is important to note that the long-term stability of this electrolyser configuration depends on the selectivity of the cellulose acetate membrane, as impurities tend to accumulate on the seawater side of the membrane, leading to increased impurity concentration and water penetration loss.

The anti-biofouling coatings will benefit the approach for long-term operation. Although the system configuration using the FO membrane was innovative, the FO membranes are not completely selective. Therefore, Cl^−^ passing through the membrane will be oxidized to Cl_2_ (lower pH) or hypochlorite (higher pH) at the anode, and the products can damage the electrolyser and electrode. At a high current density, Cl^−^ on some electrode materials will also be oxidized to chlorate and perchlorate, which requires additional removal processes from seawater. The placement of the FO membrane is also inappropriate because the small contact area between the FO membrane and water results in insufficient water feed when the electrolyser is operated at a high current density. Subsequently, Nocera et al. modified the system by placing a low-cost semipermeable membrane between the electrodes to separate the generated gas. This approach not only reduced the cost of the membrane but also minimized Cl^−^ oxidation at the anode [89].

Xie et al. have innovatively utilized a PTFE-based hydrophobic and breathable membrane as the gas-liquid interface, coupled with a concentrated KOH solution for the Self-Diffusion Electrolyser (SDE), to realize an apparatus that integrates in situ water purification with seawater electrolysis based on a self-driven phase-change mechanism (Figure 8b,c) [28]. This configuration allowed for the diffusion of water vapour while completely preventing the penetration of liquid seawater and impurity ions. The difference in vapour pressure between the seawater and the SDE causes the seawater to evaporate spontaneously. The resulting steam then passes through the membrane and condenses back into liquid form in the SDE. The consumption of water in the SDE through electrolysis maintained the pressure differential across the membrane, ensuring the continuous ingress of freshwater. The hydrophobic porous PTFE membrane introduced a tightly coupled micrometer-level gas diffusion pathway between the seawater and SDE, to direct the transfer of water vapour and effectively prevent the permeation of liquid. The polytetrafluoroethylene structure’s low surface energy formed an ultra-hydrophobic barrier that suppresses the permeation of seawater and ions over time. They established a model to balance water migration with the water consumption of electrolysis.

The results showed that by adjusting the concentration and current values of the SDE while keeping the seawater concentration constant, continuous and stable H_2_ production could be achieved. The system’s static equilibrium performance was confirmed through multiple cycle experiments, demonstrating sustainable cyclic capacity and the potential for stable electrolysis. When water migration caused by the interface pressure difference and the water consumption by electrolysis reached a dynamic equilibrium per unit time, the system was able to provide a stable “in situ water purification-electrolysis” process, enabling the continuous and efficient production of H_2_ from seawater. The system worked efficiently and stably for over 3200 h at a current density of 250 mA cm^−2^ and a voltage of 2.1 V. This serves as an excellent example of retrofitting commercial AWE for sustainable seawater electrolysis.

However, some limitations are also noticed for AWE electrolysers in practical applications [90]:

(i) Low working current density: AWE operates at a lower current density, typically limited to 0.6 A cm^−2^ at the maximum, resulting in lower production and energy efficiency. Due to the high internal resistance of the electrolyte, the energy consumption for hydrogen production can be as high as 5–7 kWh/m^3^ H_2_. The produced hydrogen gas is approximately 99.7% pure but contains residual alkali, necessitating further purification.
(ii) Slow response rate: Rapid shutdown or startup of AWE cells is challenging, leading to the difficulties in adjusting the H_2_ production rate quickly. When starting up, the cell temperature is initially insufficient for H_2_ production, with the consumed power used primarily to generate heat and raise the cell temperature.
(iii) Reaction of Alkaline Electrolyte with CO_2_: Alkaline electrolytes, such as KOH, react with CO_2_ in the air, forming insoluble carbonates under alkaline conditions. These insoluble carbonates block the porous catalytic layer, hindering the transfer of reactants and products, and significantly reducing the performance of the electrolysis cell.
(iv) Difficulty in integration with off-grid renewable energy: The slow response rate of alkaline electrolysis cells and challenges in rapid shutdown or startup, combined with the characteristics of the materials used inside the cell, means that the operational power cannot fall below a certain threshold to avoid the risk of hydrogen and oxygen crossover exceeding the explosion limit. Therefore, it is difficult to independently pair with renewable energy generation in off-grid scenarios without installing electrochemical storage or adding fuel cells to adjust the load.
(v) Large volume: The current density of alkaline electrolysis cells at atmospheric pressure is 0.2 A cm^−2^ and 1 A cm^−2^ under pressure, which is lower compared to other electrolysis cell designs. This necessitates a larger surface area for the same power output, resulting in larger cell volumes.

It is necessary to develop more universally applicable and energy-efficient electrolytes to be adapted to different environmental conditions for seawater electrolysis. The following part will discuss other electrolysis cells and seawater electrolysis solutions currently under investigation.

### 3.2. PEMWE Electrolyser

The schematic illustration of the PEMWE electrolyser is shown in Figure 6c, where proton (H^+^), in the form of H_3_O^+^, migrates to the cathode through PEM to form H_2_ [91,92,93,94]. Owing to the compact structure, the interelectrode distance is minimized between the electrodes via the thin PEM, hence, reducing ohmic polarization, which directly lowers the working voltage and energy consumption in the operating current density. This design also effectively separates H_2_ and O_2_ gases to achieve higher purity of the product gases. PEMWE exhibits a faster dynamic response than the AWE electrolyser, which enables it to be adapted to the variability of renewable energy generation and meets the requirements of off-grid electricity production.

**Figure 9:**
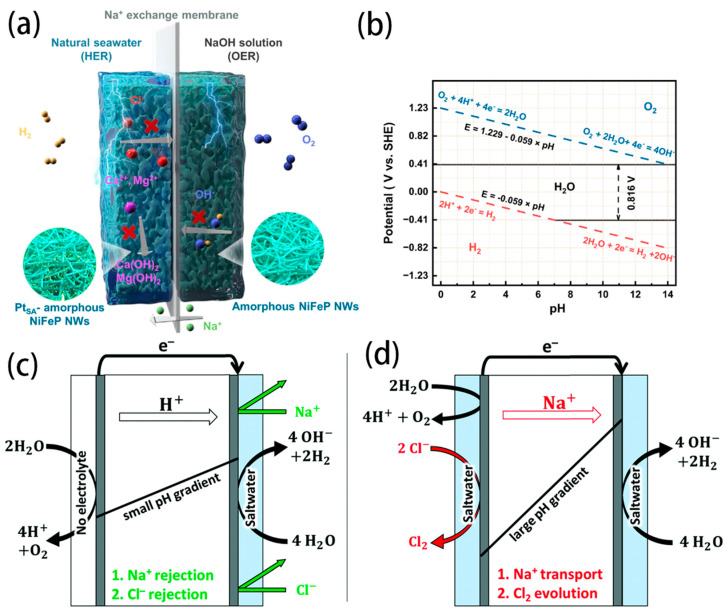
(**a**) Scheme for the asymmetric electrolyser with Na^+^ exchange membrane. (**b**) The pourbaix diagram of water [19]. Copyright 2023, Nature. (**c**) Water electrolyser using a vapor feed at the anode and saltwater at the cathode and (**d**) water electrolyser using saltwater at the anode and the cathode [20]. Copyright 2021, Royal Society of Chemistry.

Directly using seawater for electrolysis with a pure water PEM electrolyser presents some problems. In the PEMWE electrolyser, the anode needs to work in an acidic environment, which is a challenge for the OER selectivity in seawater electrolysis. Otherwise, the formation of Cl^−^ and other ClO_x_ in an acidic environment is favorable to corroding membranes, catalysts, bipolar plates, and other related accessories, reducing the efficiency and elongating the lifetime of electrolysers. In analogy to the PEMWE, a pH-asymmetric electrolyser with a Na^+^ exchange membrane is designed for seawater electrolysis, as shown in Figure 9a [19]. The natural seawater is circulated in the cathode chamber, while the NaOH solution is circulated in the anode chamber. An appropriate flow rate at the cathode can maintain pH < 9.5 to alleviate the Ca^2+^/Mg^2+^ precipitation. Simultaneously, the Na^+^ exchange membrane can prevent Cl^−^ from passing through to the anode and avoid undesired ClOR. Due to the different pH values of the two electrodes, as shown in Figure 9b, the required voltage decreased from 1.23 V to 0.82 V for the seawater electrolysis. This could be harnessed to reduce the energy cost of hydrogen production. Consequently, the asymmetric electrolyser exhibits current densities of 10 mA cm^−2^ and 100 mA cm^−2^ at voltages of 1.31 V and 1.46 V, respectively. It can also reach 400 mA cm^−2^ at a low voltage of 1.66 V at 80 °C.

A new approach is proposed to minimize Cl^−^ evolution and improve OER performance by using a humidified gas stream (no liquid electrolyte) for the anode and a liquid saltwater catholyte (Figure 9c,d) [20]. Charge repulsion of Cl^−^ by PEM resulted in low Cl^−^ generation, with anodic faradaic efficiencies for OER of 100 ± 1% with synthetic brackish water (50 mM NaCl, 3 g L^−1^) and 96 ± 2% with synthetic seawater (0.5 M NaCl, 30 g L^−1^). The enhanced H^+^ transport by the electric field enabled more efficient pH control across the cell, minimizing Na^+^ transport in the absence of a liquid anolyte. The vapor-fed anode configuration showed similar performance to a conventional PEM electrolyser up to 1 A cm^−2^ when both anode and cathode were fed with deionized water. Much lower overpotentials could be achieved by using the vapor-fed anode compared to a liquid-anolyte, as shown by adding NaClO_4_ to the electrolytes.

The PEMWEs mentioned above may have some limitations. One limitation is that the exchange performance of the membrane will decrease with the long-term operation due to the other impurity cations that exist. Another limitation is the vapour inlet requires a significant number of heat to boil seawater to the vapor phase.

### 3.3. AEMWE Electrolyser

The configuration of the AEMWE electrolyser is depicted in Figure 6b, where AEM is strategically positioned between the cathode and anode [95,96]. Water is introduced into the electrolyser from the cathode, while an inert gas is fed into the anode to carry away the produced O_2_ and H_2_O vapor. Operating at high pH conditions can minimize the oxidation of Cl^−^, making AEMWE more attractive for direct seawater electrolysis. The AEMWE with asymmetric electrolyte feeds was first designed by Strasser et al. to operate directly on natural seawater. This approach enables direct feed of neutral seawater at the cathode in a single pass, while circulating pure KOH electrolyte at the anode [97].

**Figure 10:**
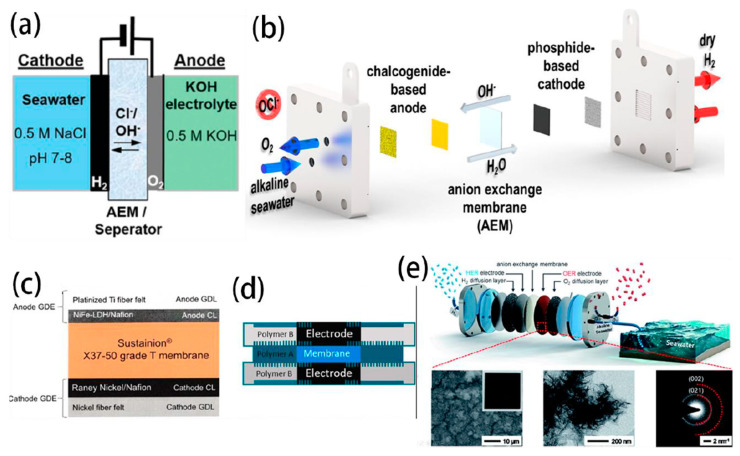
(**a**) AEMWE electrolyser configured with asymmetric feeds [97]. Copyright 2020, Royal Society of Chemistry. (**b**) schematic of AEMWE configuration on alkaline seawater electrolysis [98]. Copyright 2023, American Chemical Society. Schematic representing (**c**) the cell configuration (**d**) the cell electrode assembly [22]. Copyright 2023, Elsevier. (**e**) schematic illustration of hydrogen production by AEMWE electrolyser in practical alkaline seawater [21]. Copyright 2021, Royal Society of Chemistry.

In Cl^−^-contained alkaline electrolyte, NiFe-LDH showed superior catalytic activity and OER selectivity compared to Ir-based benchmark catalysts. All Pt-group metal-free catalysts and AEM electrolysers are coupled to realize the direct seawater splitting with an industrial current density of up to 1 A cm^−2^ below 2.0 V_cell_ (Figure 10a,b) [98]. Bliznakov et al. reported over 1000 h of extended operation for advanced AEMWE (Figure 10c,d), operating directly with seawater [22]. To fabricate the membrane electrode assemblies, catalyst-coated electrodes are assembled with Sustainion^®^ X37-50 grade-T AEMs. The anode was fabricated by spraying an ink prepared from nanostructured NiFe-layered double hydroxide catalyst that was synthesized by the solvothermal method and deposited directly onto platinized titanium porous transport layers. The cathode was prepared by spraying an ink prepared from a commercial Raney-Nickel catalyst onto a nickel fibre felt. The fabricated AEM electrolyser realized 1000-h operation at a constant current density of 300 mA cm^−2^. Besides, Park et al. used Ni-FeOOH as an anode and Pt/C as a cathode to assemble the AEMWE electrolyser for seawater electrolysis (Figure 10e), which showed a high current density of 729 mA cm^−2^ at 1.7 V with a high system efficiency of 76.35% [21]. Additionally, they developed a NiFeCo-LDH electrocatalyst used in the AEMWE, which exhibited remarkable OER activity (0.84 A cm^−2^ at 1.7 V_cell_) and high efficiency (77.6% at 0.5 A cm^−2^) for seawater electrolysis, outperforming the benchmark IrO_2_ electrocatalyst and meeting the Department of Energy (DOE) 2020 cell efficiency target of 77% [23]. Zhao et al. reported a cooperative B-V co-doped Ni_2_P electrode (B, V-Ni_2_P) applied as the cathode in both AWE and AEMWE [40]. Remarkably, the AEMWE delivered a stable performance, achieving 500 and 1000 mA cm^−2^ current densities at a cell voltage of 1.78 and 1.92 V, respectively.

**Figure 11:**
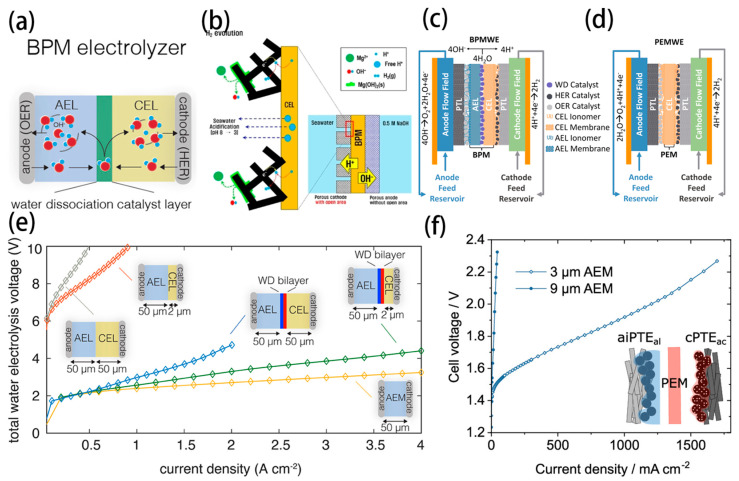
(**a**) Scheme of water transport in BPM electrolysers [99]. Copyright 2020, American Chemical Society. (**b**) Scheme of the hydroxide precipitation alleviated by the BPM designs [100]. Copyright 2022, Wiley. Cross−sectional schematic of a zero−gap (**c**) BPMWE and (**d**) PEMWE [101]. Copyright 2023, Cell. (**e**) BPM and AEMWE electrolyser reference polarization curves [99]. Copyright 2020, American Chemical Society. (**f**) Structure and performance of a BPMWE MEA with the different thickness AEM impregnation layer at the anode [102]. Copyright 2020, American Chemical Society.

The scheme of the bipolar membrane (BPM) water electrolyser is depicted in Figure 11a [99]. The suitable water dissociation catalyst is positioned at the junction between the cation-exchange layer (CEL) and the anion-exchange layer (AEL), which can efficiently dissociate H_2_O into H^+^ and OH^−^ and then migrate to the cathode and anode, respectively. The optimization of the local pH environment surrounding a membrane electrode is critical for simultaneously enhancing the kinetics of anodic reactions while circumventing chloride oxidation and the precipitation of Ca^2+^/Mg^2+^. BPM facilitates the coupling of distinct pH environments within a singular electrolyser, allowing for the independent selection of optimal pH conditions for each half-reaction. This integration holds significant promise for advancing the efficiency of the electrolytic processes.

Han et al. first reported a seawater electrolyser combined with a BPM as a separator for controlling inorganic precipitates on the cathode (Figure 11b) [100]. Despite the formation of inorganic deposits on the front side (facing bulk seawater) of the porous cathode due to the water reduction reaction, the back side facing the cation-exchange layer of the BPM remained free from thick inorganic deposits. This was ascribed to the locally acidic environment generated by proton flux from water dissociation at the BPM, enabling stable hydrogen production through proton reduction at low overpotential. This asymmetric HER at the porous cathode led to a considerably lower cell voltage and higher stability than that achieved with the mesh electrode. Moreover, precipitation at the front side of the porous cathode was further mitigated through acidification of the seawater by introducing an open area of the BPM that was not in contact with the porous cathode, allowing free protons that were not involved in the electron transfer reaction to diffuse out into the bulk seawater.

Jaramillo’s group designed, compared and evaluated PEMWEs and BPMWEs operating under asymmetric and symmetric saline electrolyte conditions to generate H_2_ and O_2_ at high current densities (Figure 11c,d) [101]. Despite higher operating voltages than PEMWEs, BPMWEs provided the combined advantages of mitigating undesired ion transport. BPM electrolysers limit the oxidation of Cl^−^ to corrosive OCl^−^ at the anode to a Faradaic efficiency (FE) of 0.005%, while PEM electrolysers under comparable operating conditions exhibit up to 10% FE to Cl^−^ oxidation. The effective mitigation of Cl^−^ oxidation by BPM electrolysers underpins their ability to enable longer-term seawater electrolysis than PEM assemblies.

Considering the configuration of BPMs, two aspects are critically important: ensuring an adequate water supply to the bipolar interface to prevent membrane drying and stalling of the reaction, and the ratio of AEM to PEM thickness, which significantly influences the performance of the electrolyser. As a potential solution, Oener et al. demonstrated that a thin Cation-Exchange Layer (CEL) could enable high-current-density BPMWE through improved water transport (Figure 11e) [99]. Similarly, Mayerhofer et al. suggested that a thinner AEL could enhance the efficiency of BPMWE, considering that proton migration within the PEM occurred 2~8 times faster than OH^−^ transport through the AEM (Figure 11f) [102]. Furthermore, the instability and high overpotential requirements of BPMs pose significant challenges for their practical applications. To address these issues, there is a pressing need to develop efficient strategies that can enhance the polarization process of water molecules, thus advancing the BPM technology towards more viable and effective real-world applications.

### 3.4. SOEC

**Figure 12:**
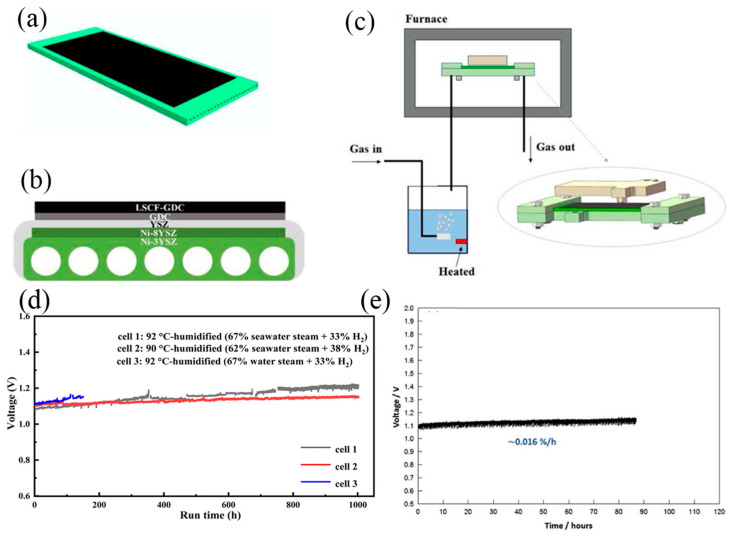
(**a**) Schematic of flat-tube solid oxide electrolysis cell; (**b**) Cross-sectional structure of the electrolysis cell; (**c**) schematic of the electrolysis test system [25]. Copyright 2023, Elsevier. (**d**) Long-term electrolysis voltages of cells with different temperatures and seawater steam content [24]. Copyright 2023, Royal Society of Chemistry. (**e**) Durability test for the contaminated cell at 0.8 A cm^−2^ and 800 °C [103]. Copyright 2017, Elsevier.

The configuration of the SOEC is illustrated in Figure 6d, where both electrodes are composed of Ni. The operating principle of SOEC involves introducing high-temperature seawater steam into the cathode, where it gains electrons to produce H_2_ and ionizes to form O^2−^. The O^2−^ ions conduct through the electrolyte to the anode to be oxidized into O_2_. SOEC typically operates at temperatures ranging from 600800 °C, offering higher electric energy conversion efficiency compared to room-temperature water electrolysis. Chan et al. developed steam electrolysis using an SOEC. The electrochemical performance was similar when using steam produced from pure water and seawater, and there was no significant difference in the mid-term and short-term performance. The durability test showed the degradation rate is about 16% 1000 h^−1^ (Figure 12e) [103]. Since 2020, the feasibility of SOEC was further tested for seawater electrolysis by Guan’s group. The SOEC coupled with a water bath inlet configuration is shown in Figure 12a–c. They compared the effect of different steam/hydrogen ratios and found SOEC trial system could work stable for over 1000 h at seawater steam contents of 62 and 67% (Figure 12d), with degradation rates of 48 mV kh^−1^ and 129 mV kh^−1^, respectively [24,25]. They also discovered that the content of Cl^−^ and Mg^2+^ decreased in the waste steam composition after seawater electrolysis, compared to the seawater before electrolysis. This observation suggests that Cl^−^ and Mg^2+^ might remain inside SOEC, potentially affecting its performance. The degradation of different seawater steam contents was mainly localized in the fuel electrode and is attributed to the migration, agglomeration, and loss of Ni particles on Ni-electrodes.

## 4. Summary and Perspectives

In summary, direct seawater electrolysis is expected to be an energy-saving technology compared to the electrolyser coupled with a desalination process. Several electrolysers have been applied to the seawater electrolysis. In terms of cost and lifetime, the AWE electrolyser is preferential for seawater electrolysis. However, due to its slow response rate, the AWE electrolyser is not very suitable for on-site seawater electrolysis using intermittent renewable energy (wind or solar energy). The PEMWE and AEMWE are designed to improve the adaptation in various situations. These electrolysers, owing to their compact structures, can decrease the work voltage to save energy. However, the PEMWE still have some limitations, such as the high-cost components, the membrane degradation and low H^+^ conductivity in the alkaline condition. Similar to the PEMWE, AEMWE also suffers from low OH^−^ conductivity, a short lifetime and membrane degradation. BPMWE is an ideal electrolyser to alleviate the problems mentioned above, but the working voltage is relatively high at the same current density condition because of the double layers of the ion exchange membrane. Lastly, pure water electrolysis, coupled with a membrane-based in situ desalination configuration, has excellent performance. In particular, Xie’s group couples the electrolyser with a PTFE-based membrane and operates stably over 3200 h.

Despite tremendous efforts made in the development of electrode materials and electrolyser designs, numerous challenges still remain to be addressed. This ongoing pursuit necessitates a continuous refinement of existing technologies and innovative approaches to overcome the inherent complexities associated with seawater electrolysis. We present some perspectives on future developments in seawater electrolysis:

1. Integration with renewable energy: Seawater electrolysis systems are being tailored to work seamlessly with renewable energy sources like solar, wind, and wave power. This integration could lead to the creation of self-sustaining ‘green hydrogen’ production platforms that operate offshore or on remote coastlines, tapping directly into the abundance of seawater and renewable energy.
2. Advanced electrolyser designs: Innovative designs that enhance the efficiency and durability of electrolysers are in development. This includes optimizing electrode materials to withstand the corrosive nature of seawater, and designing advanced membrane technologies that can handle the complex chemistry of seawater electrolysis, including the management of chloride ions.
3. Catalytic efficiency: A primary focus is to enhance the efficiency of OER and HER in seawater electrolysis. Research is ongoing into non-noble metal catalysts and novel alloy compositions that could provide similar or better efficiency at a lower cost and with higher durability.
4. Selective ion separation: Technological advancements in selective ion membranes or separators can prevent the formation of harmful byproducts such Cl^−^. These components are crucial for increasing the viability of the process, especially at the high current densities required for industrial-scale hydrogen production.
5. Scale-up challenges: The scale-up of seawater electrolysis faces challenges, which causes future research directed at maintaining efficiency and stability while scaling up the operation to meet commercial and industrial demands.
6. Environmental and economic viability: The development of seawater electrolysis technology is not only a technical challenge but also an environmental and economic one. It is critical for addressing the potential environmental impacts and ensuring the economic competitiveness of hydrogen production from seawater electrolysis. Life cycle assessments and cost analyses are integral to this effort.
7. Regulatory and safety standards: With the technology development, it is essential to establish regulatory and safety standards, which will include guidelines for the installation, operation, and maintenance of large-scale seawater electrolysis plants, particularly in off-grid environments.

In summary, although significant progress has been achieved in the field of seawater electrolysis, advanced technologies remain to be improved by innovation and collaboration to overcome current challenges.

## Figures and Tables

**Table 1:** Comparison of different hydrogen types.

	Gray Hydrogen	Blue Hydrogen	Green Hydrogen
Process	Reforming or gasification	Reforming or gasification with carbon capture	Electrolysis
Energy sources	Fossil fuels	Fossil fuel	Renewable electricity
Estimates of emission from the production process	Reforming: 9–11 Gasification: 8–20	0.4–4.5	0

**Table 2:** Comparison of four main seawater electrolysers.

	AWE	PEM	AEM	SOEC
Electrolyte	KOH 5–7 mol L^−1^	PFSA membranes	DVB polymer support with KOH or NaHCO^3^ 1 mol L^−1^	Yttria-stabilized Zirconia (YSZ)
Separator	ZrO_2_ stabilized with PPS mesh	Solid electrolyte	Solid electrolyte	Solid electrolyte
Electrode/Catalyst (Anode)	Nickel coated stainless steel	Iridium oxide	Nickel or NiFeCo alloys	Perovskite-type
Electrode/Catalyst (Cathode)	Nickel coated stainless steel	Platinum nanoparticles	High surface area nickel	Ni/YSZ
Bipolar plate	Nickel coated stainless steel	Platinum-coated titanium	Nickel-coated stainless steel	none
Sealing	PSU, PTFE	PTFE, PSU, ETFE	PTFE, Silicon	Ceramic glass
Current density	<0.6 A cm^−2^	0–4 A cm^−2^	0.2–2 A cm^−2^	0.2–0.4 A cm^−2^
Power consumption	4.3–5.7 kWh/Nm^3^	5.8–7.3 kWh/Nm^3^	5.2–4.8 kWh/Nm^3^	-
H_2_ purity	>99.8%	>99.99%	>99.99%	-
Work temperature	65–100 °C	50–95 °C	50–85 °C	600–800 °C
Cell Pressure	25–30 bar	<30 bar	<35 bar	-
Advantages	Well established; Low cost	Immediate response; High current densities; High H_2_ purity	PGM-free electrocatalysts; Low corrosion;	High electricity efficiency; Security; No pollution;
Disadvantages	Low current densities; Alkaline corrosion; Expensive maintain cost; Low H_2_ purity; High gas crossover;	Acid corrosion; Noble metal catalysts; Highly cost components;	Low OH^−^ conductivity; Short lifetime; Membrane degradation; High catalyst loading;	High temperature; Laboratory stage
Development status	Small scale application	R&D	R&D	R&D
